# Landmarks in the Evolution of (t)-RNAs from the Origin of Life up to Their Present Role in Human Cognition

**DOI:** 10.3390/life6010001

**Published:** 2015-12-23

**Authors:** Darko Balke, Andreas Kuss, Sabine Müller

**Affiliations:** 1 Institute for Biochemistry, Ernst-Moritz-Arndt-University Greifswald, Felix-Hausdorff-Str. 4, 17489 Greifswald, Germany; balked@uni-greifswald.de; 2 Department of Human Genetics, University Medicine Greifswald and Interfaculty Institute of Genetics and Functional Genomics, University of Greifswald, Fleischmannstr. 42-44, 17489 Greifswald, Germany

**Keywords:** tRNA, RNA world, aminoacylation, RNA modification, cognition, epigenetics

## Abstract

How could modern life have evolved? The answer to that question still remains unclear. However, evidence is growing that, since the origin of life, RNA could have played an important role throughout evolution, right up to the development of complex organisms and even highly sophisticated features such as human cognition. RNA mediated RNA-aminoacylation can be seen as a first landmark on the path from the RNA world to modern DNA- and protein-based life. Likewise, the generation of the RNA modifications that can be found in various RNA species today may already have started in the RNA world, where such modifications most likely entailed functional advantages. This association of modification patterns with functional features was apparently maintained throughout the further course of evolution, and particularly tRNAs can now be seen as paradigms for the developing interdependence between structure, modification and function. It is in this spirit that this review highlights important stepping stones of the development of (t)RNAs and their modifications (including aminoacylation) from the ancient RNA world up until their present role in the development and maintenance of human cognition. The latter can be seen as a high point of evolution at its present stage, and the susceptibility of cognitive features to even small alterations in the proper structure and functioning of tRNAs underscores the evolutionary relevance of this RNA species.

## 1. Introduction

In modern life forms, RNA is involved in a number of cellular activities. Three classes of RNA molecules, mRNA, tRNA and rRNA, have been known for a long time as players in the expression of genetic information: mRNA as the messenger that conveys information from the DNA in the nucleus to the site of protein synthesis in the cytoplasm, tRNA as the carrier of specific amino acids and rRNA as essential constituent of the ribosome, where proteins are made. Over the past three decades, the picture of RNA being just the minion of gene expression has dramatically changed. A large number of small and long non-coding RNAs have been discovered to act as catalysts (ribozymes), as modulators of molecular interactions in the cell, and most importantly, as regulators of gene expression. The functional diversity of RNA and the many RNAs involved in major cellular processes support the idea of the existence of an RNA world at the origin of life, where RNA was the primary living substance [1,2]. The hypothesis of the RNA world [3], already proposed in 1968 [4,5,6], was strongly supported by the discovery of Sidney Altman and Thomas R. Cech, who showed for the first time that RNA is capable of catalyzing chemical reactions [7,8]. For this finding, they eventually were honored with the Nobel Prize, since up to then proteins, today’s key players of the cell, were thought to be the only cellular catalysts. Another supporting piece of evidence for the RNA world is provided by “molecular fossils” that can be found in modern metabolism, such as RNA-like cofactors [9] or the fact that DNA synthesis proceeds via RNA intermediates [10]. Nevertheless, considering that proteins are involved in replication, transcription, translation and other cellular processes, in which nucleic acids depend on proteins, it seemed more likely that proteins could have evolved earlier than nucleic acids. However, with the discovery of ribozymes, suddenly a clue was found that it could have been just as well the other way around. Moreover, now RNA was likelier to have been evolved earlier than proteins since RNA is capable of both key features: storing genetic information and catalyzing chemical reactions.

Still, at some point the world of RNA must have proceeded towards higher complexity, integrating other molecular entities for storage of genetic information and gene expression. Taking a closer look at what we know today as tRNA allows us to hypothesize that transfer of amino acids onto RNA as well as incorporation of modified nucleotides in RNA (or post-synthetic modification) may have been processes that marked the rise of higher structural, and consequently functional complexity. Indeed, several papers discuss the ability of RNA to self-aminoacylate as a key event at the transition from the RNA world to DNA/protein based life, and it has been suggested that modern protein synthesis may have evolved from a set of aminoacyl transfer reactions catalyzed by ribozymes [11,12,13,14,15,16,17,18,19,20]. In the setting of the RNA world, RNAs with the capability of self-aminoacylation could have had a selection advantage because of a possibly higher functionality gained from the attached amino acid. Later, those aminoacyl–RNAs may have played the role of starting material for the synthesis of peptides, thus constituting pre-cursors of modern tRNA molecules as aminoacyl carriers. Modern tRNAs as key molecules for efficient and accurate protein translation are heavily modified post-transcriptionally, which is very important for tRNA structure, function and stability. Specific modifications in the anticodon loop can directly affect the behavior of tRNAs during translation, e.g., by influencing the codon–anticodon interactions. Other modifications serve structural and stabilizing purposes, for example by tuning the sugar conformation to increase or decrease the rigidity of the RNA backbone and thus the flexibility of the tRNA structure [21]. Hypomodified tRNAs are targeted for degradation [22], and it is becoming more and more clear that a link exists between tRNA modifications and human diseases such as cancer, mitochondrial-linked disorders or neurological disorders [23,24].

Focusing on tRNA development and modification, this review aims to highlight important stepping-stones in the fate of tRNAs from their origin in ancient RNA world organisms to their role in human brain development and function.

## 2. RNA Aminoacylation and the Origin of tRNA

**Figure 1:**
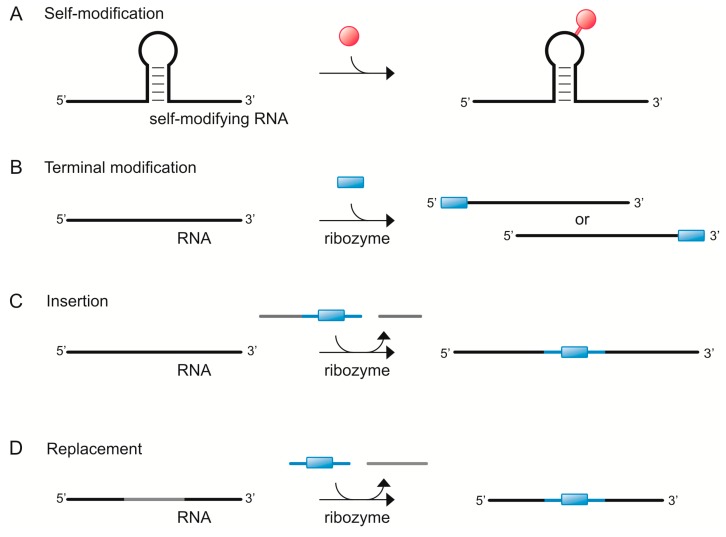
Ribozyme-mediated RNA modification: (**A**) Introduction of a modification (red circle) catalyzed by a catalytic motif within the RNA to be modified [44,45]. Note that self-modification can occur also 5′- or 3′-terminal (as for aminoacylation [13,14,15,16,17,18,25,41]); (**B**) Ribozyme mediated RNA modification of the 5′- or 3′-terminus, respectively, using modified building blocks (blue rectangle) such as nucleotide or nucleobase derivatives [57,58,59,65]; (**C**) Ribozyme mediated insertion of a short modified oligonucleotide into the target RNA [66]; (**D**) Ribozyme mediated replacement of a short RNA segment for a modified oligonucleotide [67,68,69,70].

The RNA world hypothesis describes a period in the early history of life, where RNA (or alternative nucleic acid chemistry) was the carrier of genetic information as well as the supporter of metabolic transformations [1,2]. As such, RNA is seen as a plausible precursor of today’s DNA–RNA–protein-based life. The transition of the RNA world to the complex system on which current life is based may have been characterized by RNAs capable of self-aminoacylation (Figure 1A), and indeed a number of research efforts have focused on the demonstration of this activity. Numerous ribozymes that support aminoacylation have been developed by *in vitro* selection, the first one being a small RNA that rapidly aminoacylates its own terminal 2′- or 3′-OH group with aminoacyl adenylate (aa-AMP) as activated donor [25]. Other ribozymes with similar activities followed, including a small catalytic RNA that supports the transfer of an amino acid from the 3′-end of a short RNA substrate to its own 5′-end, from amino acid cyanomethyl esters (aa-CME) or related aminoacyl donors onto the 5′-OH of a short RNA and further onto the 3′-end of a specific tRNA [13,14,15]. There are also examples of aminoacylation using nonactivated amino acids that are transferred onto the 5′-terminal triphosphate of the supporting ribozyme [20], thereby activating the amino acid for the following aminoacyl transfer onto the 3′-terminal 2′- or 3′-OH group of a suitable RNA substrate [26]. This scenario is very close to the two-step mechanism (first step: amino acid activation; second step: transfer) of aminoacylation by aminoacyl-tRNA synthetases in modern biochemistry. Some other RNAs were found to also catalyze peptide synthesis in addition to aminoacyl transfer [16,17,18,19]. A tiny ribozyme as small as five-nucleotides was found to perform RNA acylation in *trans*, thus behaving like a true enzyme, and moreover, to catalyze the formation of peptides up to a length of three amino acids [18]. Those “early” peptides in turn may have assisted RNA folding by acting as chaperones [27].

An early translation system may have used ribozymes for aminoacylation of tRNAs or tRNA-like molecules. tRNA-like molecules as precursors of modern tRNA may have consisted for example of a short aminoacyl-acceptor stem; accordingly, a minihelix-loop RNA was suggested as primordial analog of tRNA [28]. Numerous models for the origin of tRNA were proposed (reviewed in [29,30]). Several of these models are based on the assumption that tRNAs were made from two halves, one containing the CCA end, the other the anticodon [31,32,33,34,35], for example by duplication and eventually ligation of appropriate stem-loop structures [36,37]. It was hypothesized that the CCA end containing half is more ancient [32,33], which is supported by the fact that it can form a minihelix that is accepted as substrate for aminoacylation by modern aminoacyl-tRNA synthetases [38] and moreover by ribozymes [13,14]. Thus, in the RNA world, the 3′-half of the tRNA containing the CCA end was presumably sufficient for aminoacylation, and only at a later stage complemented by the 5′-half. Alternatively, tRNAs may have formed by the hybridization of small fragments to larger structures, thus initially following the principle of molecular cooperation. Later, these fragments may have become ligated.

Yet another model, the genomic tag hypothesis, suggests that minihelix RNAs containing the anticodon may have acted as tags to mark already existing ssRNA genomes for replication [39,40]. This model allows to propose a mechanism for RNA duplication/replication: two minihelix RNAs, each resembling an anticodon loop and an unpaired self-complementary tail of four nucleotides, may interact to form homodimers, with the anticodon loops at distal ends of the dimer. One anticodon pairs with the template, the other with the incoming substrates, e.g., trinucleotides, to be polymerized [27]. Taken together, the majority of models favor the concept of an evolution of tRNA from two independent RNA domains. The half of the tRNA containing the CCA end probably was an early substrate for aminoacylation, whereas the other half, which contained the anticodon loop, may have fulfilled the function of organizing other RNAs, e.g., for templated polymerization.

Nevertheless, there is also the possibility that aminoacylation catalyzed by ribozymes also occurred on more complex structures other than minihelix RNAs. In 2001, Suga and coworkers, for example, demonstrated that an *in vitro* evolved precursor tRNA, consisting of a catalytic 5′-leader sequence and an aminoacyl-acceptor tRNA, selectively self-charges an amino acid onto its 3′-terminus. Remarkably, this *cis*-active ribozyme was shown to be a substrate for RNaseP RNA to cleave the 5′-leader segment and thus to generate the mature tRNA [41]. Moreover, it was suggested that this ribozyme enhances specificity for a certain amino acid by coupling active site folding with tRNA docking, a mechanism that is used by several modern aminacyl-tRNA synthetases [42].

## 3. tRNA Modification “Then and Today”

Taking a closer look at tRNAs, one can observe that they contain a large spectrum of chemical modifications, which are necessary for correct tRNA folding and function. Modifications in particular of tRNAs and rRNAs are not unusual. According to the RNA modification database, there are 112 RNA modifications that are currently known [43]. Thus, finding out more about how RNA modification was achieved before it was taken over by proteins would help to provide additional evidence in favor of the RNA-world hypothesis. Indeed, in 1995 Wilson and Szostak reported for the first time a self-alkylating ribozyme [44] (Figure 1A). Using systematic evolution of ligands by exponential enrichment (SELEX) they developed a ribozyme able to bind the haloacetyl derivative *N*-biotinyl-*N′*-iodoacetyl-ethylendiamine (BIE) and to catalyze formation of a carbon-nitrogen bond (*N*-alkylation). Interestingly, the ribozyme shows a tRNA-like structure, leaving space for the assumption that tRNAs might have modified themselves (not surprisingly, Wilson and Szostak point out that the ribozyme could also be engineered to a *trans*-acting form). Inspired by this RNA self-modification, Sharma and co-workers generated another self-alkylating ribozyme that is able to covalently link a fluorophore to RNA [45] (Figure 1A). They showed that self-labeling with fluorescein iodoacetamide (FIA) was specific to the ribozyme sequence, which however, can be integrated into any RNA sequence to make it fluorescently detectable. These two self-alkylating ribozymes evolved by SELEX demonstrate the potential of ribozymes for RNA modification and allow hypothesizing that also tRNA modifications other than alkyl groups could have been catalyzed by ribozymes. Thus, another tessera of the mosaic of an RNA world was found.

Still, RNA modifications did not necessarily occur in a single step. Looking at protein catalysts, many enzymes first activate a compound to let it react in the second step. Mostly, ATP is used as chemical energy for this activation step. Possibly, ribozymes have also used triphosphates as activating reagent. Moretti and Müller report on a ribozyme obtained by *in vitro* selection that triphosphorylates 5′-hydroxyl groups of RNA using trimetaphosphate (TMP) [46]. The relatively small *trans*-acting ribozyme (96 nt) shows reaction rates of 0.16 min^−1^ under optimal conditions, which is in the typical range of ribozyme reaction rates. Moretti and Müller postulate that such a ribozyme could have been of significance for RNA world organisms, since RNA 5′-triphosphates could have been used for RNA polymerization [47,48], RNA–RNA ligation [49,50,51,52,53], phosphoamidite bond formation [54], RNA capping [55,56], and the activation of amino acids [20]. Possibly, this or a similar ribozyme could have also been used to activate nucleosides to be further modified.

In modern metabolism, nearly all pathways use the activated ribose 5-phosphoribosyl-1-pyrophosphate (PRPP) as substrate for nucleotide synthesis. In 1998 Unrau and Bartel selected a PRPP-dependent pyrimidine nucleotide synthase ribozyme capable of synthesizing tethered 4-thiouridine-5′-monophosphate (^4S^UMP) [57], and six years later Lau *et al.* isolated a purine synthase ribozyme that can synthesize tethered 6-thioguanosine-5′-monophosphates (^6S^GMP) [58]. Interestingly, Lau and Unrau could show that ribozyme-mediated nucleotide synthesis can also be performed with unactivated tethered PR and ^6S^GMP as substrates [59] (Figure 1B). These works demonstrate how nucleobase-modified nucleotides could have been synthesized to be subsequently incorporated into a RNA strand. Modifications of nucleotides did not necessarily have to be performed by ribozymes. However, the range of various chemical reactions that can be catalyzed by ribozymes, including aldol reaction [60], amide bond formation [61], Diels–Alder reaction [62], isomerization [63] and Michael reaction [64], support the assumption that ribozymes might have made a significant contribution.

An interesting ribozyme-based way of incorporation of modified nucleotides into a RNA strand was reported by Mutschler and Holliger, who used the hairpin ribozyme as a non-canonical 3′-5′ nucleotidyl transferase [65]. That way it was possible to add 2′,3′-cyclic AMP, GMP, UMP and CMP to the 5′-hydroxyl terminus of a RNA strand (Figure 1B). In addition, it has been shown that this reaction can also be performed with 2′,3′-cyclic phosphate-activated β-nicotinamide adenine dinucleotide, or alternatively, with ribotrinucleotides and RNA pentamers as substrates for RNA 3′-5′ extension. Furthermore, it was found that the incorporation of modified oligonucleotides into RNA can be achieved by ribozyme-mediated *trans* insertion-splicing [66]. The site-specific insertion of a RNA segment into a separate RNA substrate (Figure 1C) can be catalyzed by a group I intron derived from *Pneumocystis carinii*. Interestingly, this ribozyme accepts modified oligonucleotides, making it possible to insert sugar modified (deoxy or methoxy substitutions), backbone modified (phosphorothioate substitutions) or base modified (2-aminopurine or 4-thiouridine) segments into RNA. Another sophisticated RNA modifying strategy makes use of twin ribozymes [67,68,69]. Twin ribozymes consist of two hairpin ribozymes and thus are able to cleave and ligate a substrate RNA at two defined positions in a strictly controlled fashion, making it possible to excise a short RNA patch and replace it by a modified oligonucleotide (Figure 1D). Hence, this technique allows the incorporation of a broad range of modifications into RNA [68]. Additionally, it has been reported that twin ribozymes are potentially suited for the repair of a mutated gene at the level of mRNA [70] leading to the assumption that twin ribozymes may have been used not only for the introduction of RNA modifications but also as a kind of RNA repair system that could have been used to remove unwanted RNA modifications or mutations in the first RNA world organisms.

In today′s world, RNA modification is mainly mediated through proteins such as methyl transferases (see, e.g., [71,72]) and with the advent of the new high-throughput technologies, which brought in their wake the rise of the various “omics”-approaches (*i.e.*, the global analysis of the genome, transcriptome, proteome, *etc.*) it has also become possible to perform global mapping of methylation sites such as *N*^6^-methyladenosine (see, e.g., [73,74]) and 5-methylcytosine (m^5^C) (reviewed e.g., by [75]). This allowed uncovering m^5^C, for example, not only in coding but also noncoding RNA species, including tRNAs (e.g., [76,77,78,79,80]), where changes of the m^5^C status seem to play a role in tRNA processing and function [81].

## 4. tRNA at an Evolutionary High Point

Along with the emerging role and growing importance of tRNAs for protein biosynthesis and other cellular processes (for review see, e.g., [82]) during evolution, they also started to present a target for alterations with a detrimental effect on the cell or, at later stages, even organismic consequences. In highly complex organisms, like the human body, even comparatively subtle changes such as alterations in the modification profile of tRNAs can cause a variety of disorders (recently reviewed by Torres *et al.* [23]), ranging from mitochondrial diseases to cancer and cognitive impairment. Higher brain functions have a very complex molecular basis, and their cognitive aspects in particular represent an evolutionarily young feature. This might explain the susceptibility of man’s cognitive abilities to mutations, which have otherwise no significant impact on the plain organismic functioning of affected individuals, as can be observed in patients with non-syndromic intellectual disability (ID), *i.e.*, ID without any other accompanying clinical features (for review see, e.g., [83]). Moreover, in recent years, it has become increasingly clear that the development and functioning of the human brain is also strongly influenced by epigenetic mechanisms, which include acetylation or methylation of the histone proteins that mediate DNA packaging or methylation of nucleotides in DNA, and also in RNA. This conclusion is founded on the discovery of various ID-causing mutations in genes that play an essential role in the epigenetic modulation of nucleic acids (see, e.g., [84,85]). Such causes are, for example, mutations in the methyl-CpG binding protein 2 (MeCP2) resulting in defective DNA-methylation in individuals with Rett syndrome (reviewed e.g., by [86]). Another example is *KDM5C*. The protein encoded by this gene has been shown to act as histone demethylase where point mutations previously found in ID patients significantly reduce enzymatic activity [87,88].

**Figure 2:**
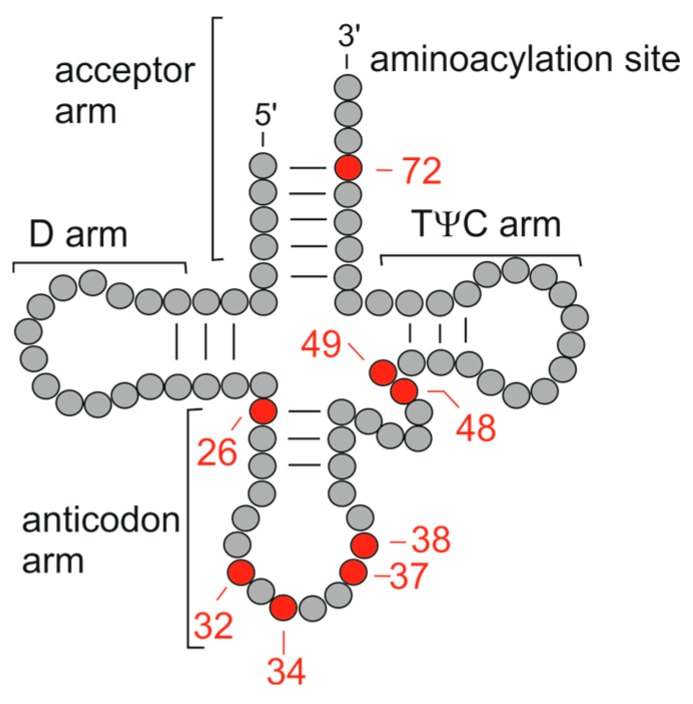
Schematic presentation of tRNA secondary structure. Nucleotide positions for modification, as mentioned in the main text, are numbered and marked in red.

By the same token, correct RNA-methylation seems to be essential for proper functioning of the brain. In higher eukaryotes, there are presently at least two prominent m^5^C RNA methyltransferases known, which are comparatively well described, and which also play a role in brain development: DNMT2 and NSUN2 [89,90]. The DNMT2 enzyme is a highly conserved protein with substantial sequence similarity to cytosine-C5 DNA methyltransferases [91]. In mice it shows strong expression during embryogenesis and was found to promote methylation of cytosine 38 (Figure 2) in tRNA^Asp^ [92]. Loss of DNMT2 function was shown to affect brain development in zebrafish [93] and double knockout of *Dnmt2* and *Nsun2* in mice revealed complementary target-site specificities for the two as well as a complete loss of cytosine-C5 methylation of tRNAs, while the phenotype included impaired thickness and a reduced level of organization in the cerebral cortex [94].

NSUN2 is an enzyme that catalyzes the intron-dependent formation of m^5^C at cytosine 34 (Figure 2) of tRNA^Leu^ (CAA) [89]. It can methylate its tRNA targets in various places, which in mice are most frequently cytosines 48 and 49 in the variable loop (Figure 2) [94]. Findings from various model organisms and cell lines suggest that NSUN2 plays a role in m^5^C RNA modification in a variety of contexts, including tissue development, differentiation and cellular signaling (e.g., [82,94,95,96,97,98]. What is more, deleterious mutations affecting the *NSUN2* gene were repeatedly found to be associated with ID [99,100,101,102], and the corresponding mouse model shows microcephaly as well as memory deficits and behavioral abnormalities [103].

NSUN2 belongs to the NOP2/Sun RNA methyltransferase family, which is characterized by containing a SUN domain that can impart interaction with Klarsicht/ANC-1/Syne homology (KASH)-domain proteins (see, e.g., [104] for review). Recently, it has been found that another member of this protein family, NSUN6 also acts as a tRNA methyltransferase in human on cytosine 72 (Figure 2) at the 3′-end of the tRNA acceptor stem [105], for which expression has been found in murine brain [106].

Additional examples for the involvement of tRNA-modifiers in the development of cognitive features are TRMT1 and FTSJ1. TRMT1 (TRM1) is a methyltransferase that dimethylates guanosines (m^2^_2_G) at position 26 (Figure 2) of tRNAs [107], and mutations were found in individuals with autosomal recessive ID from different consanguineous families [108,109]. Several mutations causing non-syndromic ID were also described for *FTSJ1* [110,111,112], whose gene product is involved in RNA-methylation as well and which is part of a duplication reported also in connection with a syndromic form of ID [113]. The FTSJ1 protein comprises 330 amino acids and is highly conserved throughout evolution. Its amino acid sequence shows 34% identity to the *Escherichia coli* (*E. coli*) heat shock protein FtsJ, which has been found to form a complex with *S*-adenosylmethionine (SAM or AdoMet) and to possess a methyltransferase fold [114]. FstJ methylates the *E. coli* 23S rRNA at position U2552, with *S*-adenosylmethionine as methyl-group donor (e.g., [115]). U2552 is a conserved part of the ribosomal aminoacyl-tRNA binding site during protein synthesis (e.g., [116]). Eukaryotic proteins with FtsJ homology are the three yeast proteins Spb1 (Suppressor of PAB1 protein 1), Mrm2 (mitochondrial methyl transferase 2) and Trm7 (tRNA methyltransferase 7). They all exhibit 2′-*O*-methyl transferase activity as well, and there is published evidence that the three homologs might localize to different cellular compartments (mitochondria, nucleolus and cytoplasm) [117,118,119]. Most recently it has also been found that cells from human carriers of deleterious *FTSJ1* mutations show an almost complete lack of Cm32 and Gm34 as well as reduced peroxywybutosine (o2yW37) (Figure 2) in tRNA^Phe^ [120].

## 5. Conclusions

The evolution of aminoacyl–RNAs from the RNA world at the origin of life to what we know today as tRNA is reflected in two characteristic features: aminoacylation and modification. Once being just a prerequisite for gain of functionality and a selection advantage, aminoacylation of tRNA in modern biochemistry is highly specific, and involves specialized enzymes, aminoacyl tRNA synthetases. Correct aminoacylation ensures proper decoding of the charged tRNA in the ribosome and translation into protein according to the genetic code. To the same extent, modification of tRNAs is directly linked to accurate and efficient translation of the genetic code, as it affects the correct charging of the tRNA by the cognate aminoacyl tRNA synthetase, decoding, tRNA folding and stability. Subtle changes in the modification state of tRNAs may have drastic effects: inaccurate decoding, improper folding and loss of function or decay by various degradation pathways. Thus, RNA modification in general and tRNA modification in particular are highly complex and fine-tuned processes of tremendous significance for life. Not least with the findings from the Encode project [121], it is becoming more and more clear that RNA species (still) play important roles on many levels in cellular and organismic functioning. Evolution as it is generally understood has so far reached a high point in the human brain. Since even in this context RNAs can be found to be involved in the development and maintenance of cognitive features, the most distinguishing and unique features of this organ that seem so far unsurpassed in the animal kingdom, it can be surmised that they took over crucial parts on every other phylo- and ontogenetic stage as well. It will thus be an important task for future RNA research to further elucidate not only the development of molecular features of RNA but also to learn more about the contribution of the RNA world to the origin and evolution of modern protein-based life as we know it today.
